# Use of *Lactiplantibacillus pentosus* O17 as a Starter Culture for the Production of *Gaeta*-like Table Olives

**DOI:** 10.3390/foods15071257

**Published:** 2026-04-07

**Authors:** Marilisa Giavalisco, Annamaria Ricciardi, Emanuela Lavanga, Attilio Matera, Nicola Condelli, Teresa Zotta

**Affiliations:** Department of Agricultural, Forestry, Food and Environmental Sciences, University of Basilicata, 85100 Potenza, Italy; annamaria.ricciardi@unibas.it (A.R.); emanuela.lavanga@unibas.it (E.L.); attilio.matera@unibas.it (A.M.); nicola.condelli@unibas.it (N.C.); teresa.zotta@unibas.it (T.Z.)

**Keywords:** *Gaeta*-like olives, Lactiplantibacillus pentosus, starter cultures, spontaneous fermentation, driven fermentation, olive microbiota

## Abstract

The Protected Designation of Origin (PDO) “*Oliva di Gaeta*” is a type of Italian fermented table olive obtained through a spontaneous fermentative process (“*Itrana*” method) driven by the indigenous olives microbiota. Although the use of starter cultures may improve the fermentative process and the quality of the final product, this has been poorly investigated for PDO *Gaeta* olives. In this study, we evaluated the use of *Lactiplantibacillus pentosus* O17 as a starter culture for the production of *Gaeta*-like olives. Three fermentations were performed: a spontaneous process (according to PDO regulation, trial A), fermentation driven by *Lpb*. *pentosus* O17 (trial B), and O17-driven fermentation combined with different brine formulation (trial C). Physicochemical properties (i.e., pH, titratable acidity, salt, and total phenolic content) and microbial population (plate counting and metataxonomy) were monitored up to 180 days. Sensory and texture profiles were evaluated in the final product. Driven fermentations (trials B and C) led faster acidification and enhanced the microbial quality of table olives without altering the organoleptic features of *Gaeta*-like olives. Our study suggests that the use of a starter culture and a different brining method could improve the microbiological quality of *Gaeta*-like olives, while preserving the traditional identity and the sensory attributes related to this PDO product.

## 1. Introduction

Fermented table olives are a traditional food that significantly contributes to the local and global economy of the Mediterranean region [1]. Based on a fermentative process, they are generally classified as *Spanish-style* (olives treated with NaOH solution to remove the bitter compounds) or *Greek-style* olives (bitterness is removed through microbial enzymatic activity; see [2,3,4,5] for more details). The microbiota of fermented olives are closely derived from those of fresh and unprocessed fruits, even if production conditions may significantly shape their composition and dynamics [5,6,7]. Lactic acid bacteria (LAB; mainly *Lactiplantibacillus plantarum* and *Lpb*. *pentosus*) and yeasts (e.g., *Candida* spp., *Pichia* spp., *Saccharomyces* spp., *Debaryomyces* spp. and *Wickerhamomyces* spp.) are the dominant members of fermented olives microbiota [3,6]. Halophilic and alkalophilic bacteria (i.e., *Marinilactibacillus* spp., *Alkalibacterium* spp., *Celerinatantimonas* spp., and *Halomonas* spp.) have also been identified in several fermentative processes [8,9,10,11,12]. Other microorganisms, including *Enterobacteriaceae* spp., *Clostridium* spp., *Pseudomonas* spp., *Staphylococcus* spp., *Vibrio* spp., and occasionally molds, may be found in the early stages of fermentation or in low-quality products (see Giavalisco et al. [3] for review). Table olives are mainly produced through natural fermentative processes (driven by the indigenous microbiota), which sometimes are difficult to control, resulting in variable and low-quality products (e.g., poor acidification, development of undesirable microorganisms, and sensory defects). The use of starter cultures may therefore provide better control of the process, ensuring a greater microbial stability and overall quality of products. Members of *Lpb*. *plantarum* and *Lpb*. *pentosus*, alone or in combinations with other LAB (e.g., *Leuconostoc mesenteroides*, *Lacticaseibacillus paracasei*) or yeasts (e.g., *C*. *boidinii*, *C*. *diddensiae*, *D*. *hansenii*, *S*. *cerevisiae*, *W*. *anomalus*) have been investigated as starter cultures for the production of several fermented olives (see Giavalisco et al. [3] for a more comprehensive description of microbial consortia and their effects on the final products), and their use was effective in preventing the growth of spoilage and pathogenic microorganisms and in improving the organoleptic properties of the final products.

In this study, we evaluated the effect of *Lpb. pentosus* O17 as a starter culture for the production of a renowned Italian fermented table olive, namely the Protected Designation of Origin (PDO) “*Oliva di Gaeta*” (EU Regulation 2016/2252 [13]). PDO *Gaeta* olives are produced using the traditional “*Itrana* method,” based on the natural fermentation of fully ripe *Itrana* cv. drupes, harvested in the Lazio and Campania regions (according to PDO specifications). The process involves initial spontaneous fermentation in water, followed by the addition of salt (7% *w*/*w*, when the pH drops to 4.5) to stabilize the process. Although the artisanal method preserves the product authenticity, it may result in variable quality and limited microbiological stability. Despite the economic relevance of PDO *Gaeta* olives within the Italian sector, the potential exploitation of starter cultures for the production of this product has been poorly investigated [14].

Therefore, in this study, we compared the spontaneous fermentation of PDO *Gaeta* olives (trial A) with two processes driven by *Lpb. pentosus* O17 (trial B and C), combined (trial C) or not combined (trial B) with a different brine formulation, to evaluate the effect of a starter culture and early salt addition on the microbial profile, chemical–physical, rheological, and sensory properties of PDO *Gaeta* olives. As trials B and C were carried out by modifying the fermentative process of PDO regulation, the resulting products are described as *Gaeta*-like olives.

## 2. Materials and Methods

### 2.1. Strain and Culture Conditions

*Lactiplantibacillus pentosus* O17 (isolated from brine of *Bella di Cerignola* olives [15]) was used as a starter culture for the production of *Gaeta*-like olives. The strain was maintained as a freeze-dried stock (11% *w*/*v* skim milk with 0.1% *w*/*v* ascorbic acid) in the Unibas Yeast and Bacteria Culture Collection (collection code: UBYBCC 005; strain number O17), Università degli Studi della Basilicata (Potenza, Italy), and routinely propagated in MRS (Oxoid, Basingstoke, Hampshire, UK) at 30 °C for 16 h before assays.

For the inoculum preparation, *Lpb*. *pentosus* O17 was cultivated (30 °C, 24 h) in MRS supplemented with 4% (*w*/*v*) NaCl to allow cell adaptation to the osmotic stress, and then standardized to a final population of 10^6^ cfu/mL, in water or brine, as described below.

### 2.2. Production Process of Gaeta-like Olives

**Figure 1:**
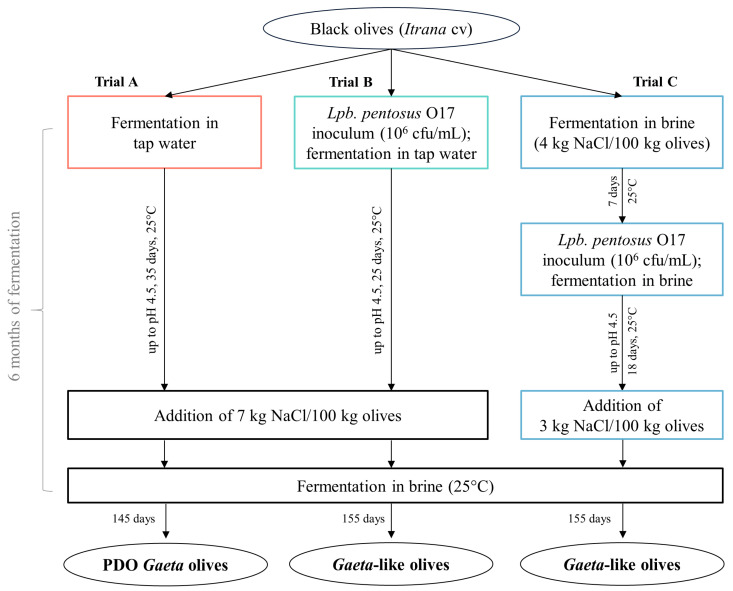
Flowchart for the production of PDO *Gaeta* olives and *Gaeta*-like olives. Trial A: spontaneous fermentation (production of *Gaeta* olives according to the PDO regulation); trial B: fermentation driven by *Lpb*. *pentosus* O17 as starter culture (*Gaeta*-like olives); trial C: fermentation driven by *Lpb*. *pentosus* O17 as starter culture and different brining formulation (*Gaeta*-like olives).

Black olives of the *Itrana* cultivar were hand-harvested (at the full ripening stage, March 2022) in olive orchards located in Formia (Lazio, Italy) and then transported to the Industrial Microbiology Laboratory, Università degli Studi della Basilicata (Potenza, Italy), for processing. The intact drupes were washed with tap water and used to produce PDO *Gaeta* olives and *Gaeta*-like olives, as described in Figure 1. A spontaneous fermentation (trial A), performed according to the production regulation for PDO *Gaeta* olives, was compared with two fermentative processes driven by *Lpb*. *pentosus* O17 (*Gaeta*-like olives). In trial B, O17 was inoculated in water (final population of 10^6^ cfu/mL) at the beginning of the process (0 days), while in trial C, the strain was inoculated in brine (final population of 10^6^ cfu/mL) after 7 days. Regarding brine formulation, in trials A and B, salt (7 kg NaCl/100 kg olives, i.e., 10.5% *w*/*v* NaCl in brine) was added when the pH reached 4.5 (as indicated in PDO regulation), while a gradual supplementation (1st step: 4 kg NaCl/100 kg olives, i.e., 6% *w*/*v* NaCl in brine at the beginning of the process; 2nd step: 3 kg NaCl/100 kg olives, i.e., 4.5% *w*/*v* in brine when pH reached 4.5) was used for trial C. Salt was completely dissolved by a magnetic stirrer. The laboratory-scale fermentations were performed in triplicate in sterile glass vessels, containing 3 kg of olives and 2 L of water (A and B) or brine (C), and incubated at 25 °C for 6 months, under anaerobic conditions.

Fermentative processes were monitored at regular interval time up to 6 months (sampling at 2, 5, 7, and 25 days for trials B and C, or 35 days for trial A, before salt addition, and then at 15, 30, 60, 90, 120, and 150 days after salt addition), by measuring pH, NaCl concentration, titratable acidity, total phenol content (TPC), and microbial population (plate counting) in both brines and olives. The composition of microbial communities (*16S rRNA* and *ITS* metataxonomy) was evaluated in brine samples after 5, 25 (trials B and C) or 35 (trial A), 60, 120, and 180 days of fermentation (microbiota of unprocessed olives was used as reference), while water activity (a_w_), moisture content, texture profile and sensory properties were evaluated in the final *Gaeta*-like olives (6 months).

### 2.3. Physicochemical Analyses

The pH of the brines and olive pastes (obtained by 10-fold dilution in water and 3 min homogenization in sterile filter bags, Stomacher 400 Lab Blender, International PBI, Milan, Italy) was measured with a CyberScan-pH110 meter (Oakton Instruments, Vernon Hills, IL, USA) Double Pore Slim electrode (Hamilton Company, Reno, NV, USA).

The titratable acidity of the brine and olive paste was measured by titration with NaOH 0.1 N, using phenolphthalein as the indicator. The pH of the solution was also checked (approximately pH 8.3). The results were expressed as gr of lactic acid/100 mL brine.

The concentration of sodium chloride in brine was measured with a chloride electrode (Mettler Toledo ISE Chloride with Crison 5241 reference electrode). An ionic strength adjuster (ISA) solution (5 M NaNO_3_) was added (2% *v*/*v*) to each sample before measuring. A standard curve was built to correlate mV values to NaCl concentration (range 0.0058–5.8 g/L).

The total phenol content (TPC) of both the brine and olive pastes (homogenized with a blender for 5 min) was measured with the Folin–Ciocâlteu assay, as described by Giavalisco et al. [16].

For the moisture content, 5 g of olive paste was placed on a previously weighted evaporating dish and dried (Selecta DigiHeat oven, J.P. Selecta, Barcelona, Spain) at 105 °C until weight stabilization (72 h). After cooling in a desiccator, dishes with the dried sample were weighted. The water activity (a_w_) of the olive paste was measured at 25 °C by using a HygroPalm hygrometer with an HC2-AW probe (Rotronic Italia Srl, Milano, Italy). All physicochemical analyses were carried out in duplicate.

### 2.4. Evolution of Microbial Profile During Fermentation

#### 2.4.1. Plate Counting

Olives were homogenized (3 min, sterile filter bags; Stomacher 400 Lab Blender, International PBI, Milan, Italy) with sterile PS (0.85% *w*/*v* NaCl + 0.1% *w*/*v* peptone; 10^−1^ dilution) and diluted serially (1:10 in PS) before plate counting; for brine samples, serial dilutions were directly performed.

Microbial counts were carried out by spiral plating (WASP Spiral Plater, bioMérieux Italia SpA, Bagno a Ripoli, Firenze, Italy) using the following substrates: (i) mMRS [17] with 100 mg/L cycloheximide and 15 mg/L nalidixic acid (mMRS + C + AN; 30 °C, 48 h, anaerobic conditions) for detecting LAB; (ii) Glucose-Yeast Extract-Agar (GYEA) with 100 mg/L chloramphenicol (GYEA + C; 30 °C, 48 h or 5 days) for yeasts and molds, respectively; (iii) Gelatin Peptone Agar (AG) with 100 mg/L chloramphenicol (AG + C; 30 °C, 48 h) for total aerobic count; and (iv) VRBGA (30 °C, 24 h) for *Enterobacteriaceae*; (v) mMRS + C + AN, or GYEA + C, or AG + C, supplemented with 6% (*w*/*v*) NaCl, were respectively used for detecting halophilic LAB, yeasts and other bacteria. At the end of incubations, colonies were enumerated using a digital colony counter (EasyCount 2, bioMérieux Italia). At the end of fermentation (180 days), *Listeria* spp. (Palcam Agar Base supplemented with Palcam Selective Supplement, Oxoid) and *Staphylococcus* spp. (Baird-Parker, Oxoid, supplemented with Egg Yolk Tellurite) were also checked.

#### 2.4.2. Metataxonomy Analysis

Metataxonomic analyses were performed to evaluate the evolution of the bacterial (V3–V4 region of *16S rRNA* gene) and yeast/fungal (*ITS2* region) microbiota in the brines (sampling at 5 and 25 days for trials B and C, or 5 and 35 days for trial A, before salt addition; at 60, 120, 180 days of fermentation). Unprocessed olives were used as the reference condition.

Total DNA was extracted using the DNeasy PowerFood Microbial kit (QIAGEN Srl, Milan, Italy), and quality and quantity were measured with a NanoDrop™ One/OneC Microvolume UV-VIS spectrophotometer (Thermo Scientific^TM^; Milano, Italy). DNA samples were sequenced on an Illumina NovaSeq platform (external service, Novogene, Cambridge, UK, July 2023); negative controls and mock community standards (ZymoBIOMICS™ Microbial Community DNA Standard, Lot number 219120; Zymo Research, Irvine, CA, USA) were included to monitor contamination and pipeline accuracy. Sequence processing, bioinformatic, and taxonomic analyses were performed by Ricciardi et al. [18]. Sequences were deposited in NCBI SRA with accession number PRJNA1034814, and the analyzed data were included in the FoodMicrobionet 5.0.1 database [19].

### 2.5. Texture Profile Analysis

The Texture Profile Analysis (TPA) was carried out on 45 olives for trial, sampling 15 olives from 3 batches. TPA parameters and units (hardness, springiness, cohesiveness, adhesiveness, gumminess and chewiness) were settled according to Lanza and Amoruso [20], with some modifications. The test performed on the lateral faces of whole drupes by using an INSTRON Texture Analyzer (Universal Instron’s 5500 Series Electromechanical Machine, Norwood, MA, USA), operating in compression mode with an SMSP/35 plate (35 mm Ø). The probe speed was set at 0.5 mm/s, with a time interval between two compressions of 5 s; the test was stopped at 25% deformation. To evaluate olive peel break force, the puncture test was carried out. The test was performed using an SMS P/1,5 needle (1.5 mm Ø) probe and a 5 kg load cell. The test speed was 1.0 mm s^−1^ and the penetration depth was limited to 4 mm. All analyses were performed at room temperature.

### 2.6. Sensory Evaluation

A panel of 12 trained judges (7 women, 5 men) was involved in the Quantitative Descriptive Analysis (QDA) of the PDO *Gaeta* olives (trial A) and *Gaeta*-like olives (trials B and C) obtained after 180 days of fermentation. Sensory attributes were generated by tasting olives from trial A in a preliminary session, in which judges expressed their consensus on the terms and definitions for odor (fermented olive, off-odor), taste (bitter, salty, fermented olive) and texture (skin hardness, ease flesh detachment from the stone) attributes. Then, each judge analyzed 3 olives per trial. Olive samples were placed in glass jars with stoppers, labeled with random 3-digit numerical codes, and presented to the judges in a balanced and randomized order. Three replicates were performed for each fermentation. The attribute intensity was reordered on a 10 mm unstructured linear scale, with anchor points at 0 (absent) and 100 (very strong).

Randomization, sample coding and test management were carried out using FIZZ software v. 2.47 (BioSystem, Couternon, France).

### 2.7. Statistical Analyses

Statistical analyses and graphs were obtained by using R 4.5.2 [21]. Analysis of Variance (ANOVA) and post hoc Tukey’s HSD (Honestly Significant Difference) test were used to estimate statistically significant differences (*p* ≤ 0.01).

## 3. Results and Discussion

### 3.1. Physicochemical Analyses of Gaeta-like Olives

*Lpb. pentosus* O17 was selected as starter culture for its phenolic compound tolerance, oleuropein degradation and hydroxytyrosol synthesis, biofilm formation, high radical scavenging activity, resistance to simulated gastro-intestinal traits and different combinations of high NaCl concentrations and low pH [15]. Additionally, preliminary survival tests (data not published), performed under simulated brine conditions (gradual addition of NaCl up to 7% *w*/*v* and olive mill wastewater up to 5% *v*/*v* to mimic the phenolic compounds diffusion from olives to brine during fermentation), demonstrated the good survival of *Lpb. pentosus* O17 (0.55 ± 0.14 log-cycle reduction) after 14 days of fermentation at 20 °C.

**Figure 2:**
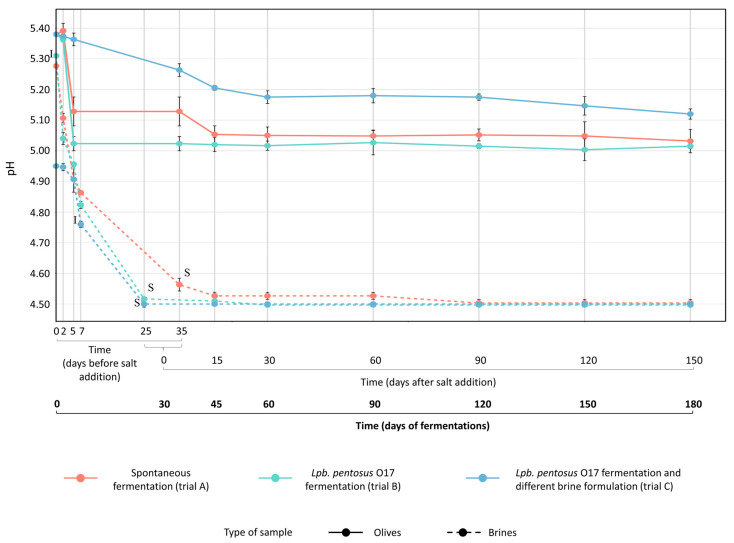
Change in pH values measured in olives (continuous lines) and brines (dotted lines) during spontaneous (trial A, red line) and driven fermentations (trial B, green line, *Lpb*. *pentosus* O17 inoculated in water at the beginning of the process; trial C, blue line, *Lpb*. *pentosus* O17 inoculated after 7 days of fermentation in 6% *w*/*v* brine solution). **I**, timing of *Lpb*. *pentosus* O17 inoculum (0 days for trial B; after 7 days for trial C). **S**, content and timing of salt addition in brine: 7 kg NaCl/100 kg olives (10.5% *w*/*v* NaCl in brine) for trials A (after 35 days) and B (after 25 days); 4 kg NaCl/100 kg olives (6% *w*/*v* NaCl in brine) at time 0 days and subsequent addition of 3 kg NaCl/100 kg olives (4.5% *w*/*v* NaCl in brine) after 25 days for trial C.

**Figure 3:**
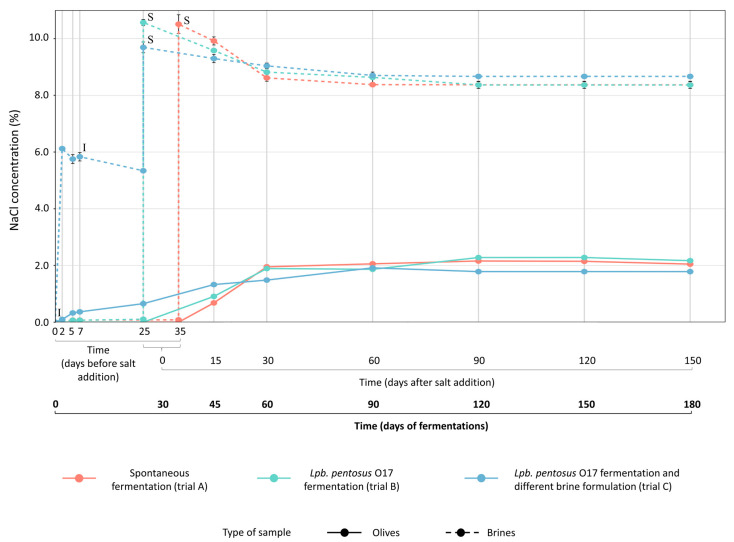
Changes in NaCl concentration measured in olives (continuous lines) and brines (dotted lines) during spontaneous (trial A, red line) and driven fermentations (trial B, green line, *Lpb*. *pentosus* O17 inoculated in water at the beginning of the process; trial C, blue line, *Lpb*. *pentosus* O17 inoculated after 7 days of fermentation in 6% *w*/*v* brine solution). **I**, timing of *Lpb*. *pentosus* O17 inoculum (0 days for trial B; after 7 days for trial C). **S**, content and timing of salt addition in brine: 7 kg NaCl/100 kg olives (10.5% *w*/*v* NaCl in brine) for trials A (after 35 days) and B (after 25 days); 4 kg NaCl/100 kg olives (6% *w*/*v* NaCl in brine) at time 0 days and subsequent addition of 3 kg NaCl/100 kg olives (4.5% *w*/*v* NaCl in brine) after 25 days for trial C.

**Figure 4:**
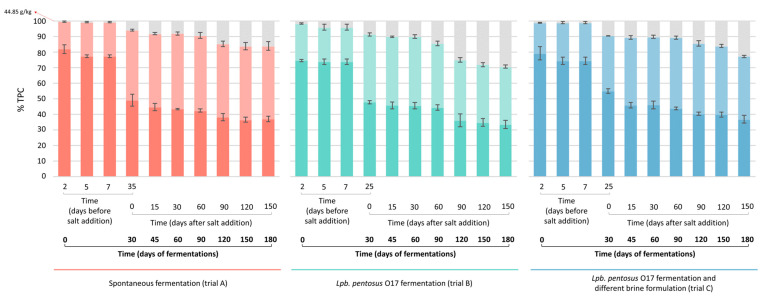
Percentage (%) of total phenolic content (TPC) measured in olive fruits (dark colors) and brines (light colors) during spontaneous (trial A, red bars) and driven fermentations (trial B, green bars, *Lpb*. *pentosus* O17 inoculated in water at the beginning of the process; trial C, blue bars, *Lpb*. *pentosus* O17 inoculated after 7 days of fermentation in 6% *w*/*v* brine solution). Gray bars: % of phenolic compounds potentially consumed or transformed by microbial metabolism. Red arrows indicate the initial values of TPC (expressed as g GAE/kg olives) in fresh *Itrana* olives.

The pH of brines changed according to the type of fermentation (Figure 2). The presence of starter boosted the pH decrease in trials B and C (reaching pH 4.5 after 25 days), while spontaneous fermentation (trial A) exhibited a slower acidification trend (pH 4.5 after 35 days). Fifteen days after salt addition (45 days of total fermentation), the pH of brines remained constant at 4.5 (Figure 2, Table S1) for all trials. The pH of olives, although higher, had a similar trend to that of brines (Figure 2). According to the pH reduction, the titratable acidity in brines (Figure S1) increased in the first phase of fermentation (0–25 days for trials B and C; 0–35 days for trial A) as a result of LAB growth. As expected, *Lpb*. *pentosus* O17 (trials B and C) enhanced brine acidification compared to spontaneous fermentation (trial A). After 30 days from salt addition (60 days of total fermentation), the titratable acidity of brines slightly decreased, probably due to the reduction in the LAB population and the concurrent dominance of yeasts. This study confirmed that the use of starter cultures belonging to the *Lactiplantibacillus* group promotes a faster reduction in pH due to the production of lactic acid from fermentable sugars. The decrease in pH may also inhibit the growth of spoilage microorganisms, improving the microbiological quality and shelf-life of the final product [5,6,7,22,23,24]. The salt content in brines progressively decreased during fermentation due to the partial diffusion into the olives. After 60 days of fermentation, the salt concentration in both brines and fruits remained constant. For all trials, salt concentration was maintained at 8% (*w*/*v*) NaCl in brines and at 2% (*w*/*w*) NaCl in fruits up to the end of the processes (Figure 3). During fermentation, the TPC of olives decreased because of partial diffusion into the brine and, consequently, the phenolic concentration in the brines increased. Part of the phenolic compounds, however, was probably consumed and/or transformed by microbial activity. After salt addition, TPC variations were less marked in both olives and brines (Figure 4).

It has been widely demonstrated that *Lactiplantibacillus* species (mainly *Lpb*. *plantarum* and *Lpb*. *pentosus*) are able to hydrolyze and metabolize phenolic compounds through the activity of several enzymes (i.e., β-glucosidase, tannase, esterase, phenolic acid decarboxylases, vinyl phenol reductase, hydroxycinnamate reductase, gallate decarboxylase [25,26]). This ability, however, is strain-specific and is suitable for the selection and competitiveness of starter cultures. Some phenolic compounds, in fact, may induce significant stresses (e.g., oleuropein by affecting the structure of peptidoglycan and the cell membrane), resulting in growth inhibition and cell death [25,27].

Degradation of oleuropein is also important for the final quality of fermented olives. Perpetuini et al. [14] reported the debittering ability of two *Lpb*. *pentosus* strains used as starters for the production of *Itrana* table olives, as demonstrated by a decrease in oleuropein, demethyloleuropein, and 2-(3,4-hydroxyphenyl) ethyl (3S,4E)-4-formyl-3-(2-oxoethyl) hex-4-enoate (3,4-DHPEA-EDA) in olive flesh, with a concurrent increase in their hydrolysis products, including hydroxytyrosol. The reduction in TPC may also be attributed to the enzymatic activity of indigenous yeasts. This is consistent with previous data highlighting the significant role of yeasts (in addition to LAB) in the debittering process of table olives [28,29].

Overall, the evolution of the main physicochemical parameters (e.g., pH, TA, salt) measured in this study was consistent with previous data on the fermentation processes of *Greek-style* olives driven by LAB (e.g., *Lpb*. *plantarum*, *Lpb*. *pentosus*, *Lacticaseibacillus paracasei*, and *Lcb. rhamnosus* [13,29,30,31,32,33]).

### 3.2. Microbiological Analyses of Gaeta-like Olives

During fermentation, the evolution of total aerobic count, LAB, yeasts, molds, and *Enterobacteriaceae*, as well as halophilic LAB, yeasts, and other bacteria, was monitored in both brines and olives. The microbial population of fresh *Itrana* olives (washed before fermentation process) consisted of LAB and yeasts both at 2.3 log cfu/g, molds at <1.0 log cfu/g, total aerobic count at 4.4 log cfu/g and *Enterobacteriaceae* at 4.9 log cfu/g.

**Figure 5:**
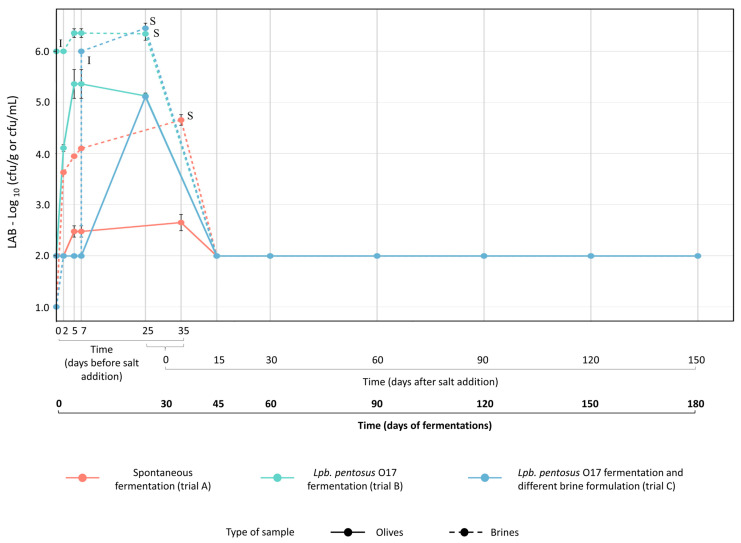
Lactic acid bacteria (LAB) populations measured in olives (continuous lines) and brines (dotted lines) during spontaneous (trial A, red line) and driven fermentations (trial B, green line, *Lpb*. *pentosus* O17 inoculated in water at the beginning of the process; trial C, blue line, *Lpb*. *pentosus* O17 inoculated after 7 days of fermentation in 6% *w*/*v* brine solution). **I**, timing of *Lpb*. *pentosus* O17 inoculum (0 days for trial B; after 7 days for trial C). **S**, content and timing of salt addition in brine: 7 kg NaCl/100 kg olives (10.5% *w*/*v* NaCl in brine) for trials A (after 35 days) and B (after 25 days); 4 kg NaCl/100 kg olives (6% *w*/*v* NaCl in brine) at time 0 days and subsequent addition of 3 kg NaCl/100 kg olives (4.5% *w*/*v* NaCl in brine) after 25 days for trial C.

During the spontaneous fermentation, the LAB population in brine increased up to 4.66 ± 0.10 log cfu/mL within the first phase of incubation (35 days, Figure 5). As expected, the LAB population in brines was affected by *Lpb*. *pentosus* O17 inoculum, and the highest level of LAB was found in driven fermentations (trials B and C) compared to the spontaneous one (trial A). In all trials, salt addition reduced the viability of LAB (>4 log cycles for trials B and C and >2 log cycles for trial A) within 15 days. The number of cells potentially adherent to the olive surface was higher in driven fermentations and increased over time up to 25 days (Figure 5). However, after salt addition, colonies attributable to *Lactiplantibacillus* strains disappear on mMRS plates for both brines and olives. Competition with yeasts may also affect the growth of LAB, including *Lpb*. *pentosus* O17.

**Figure 6:**
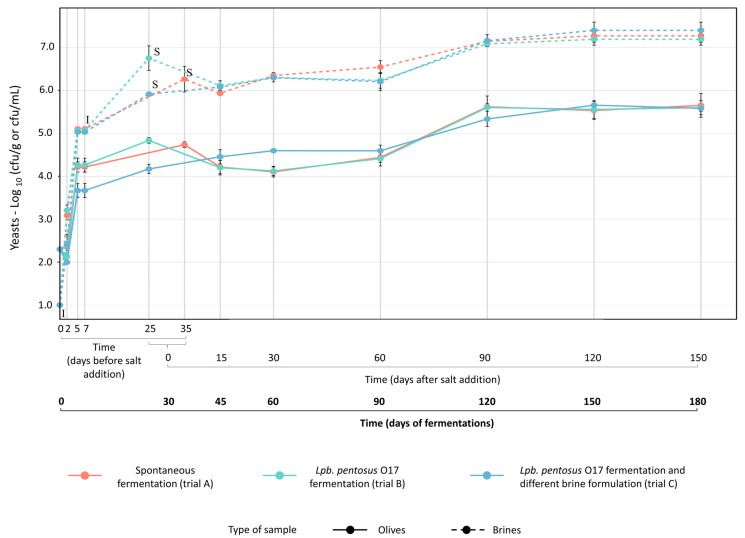
Yeast population measured in olives (continuous lines) and brines (dotted lines) during spontaneous (trial A, red line) and driven fermentations (trial B, green line, *Lpb*. *pentosus* O17 inoculated in water at the beginning of the process; trial C, blue line, *Lpb*. *pentosus* O17 inoculated after 7 days of fermentation in 6% *w*/*v* brine solution). **I**, timing of *Lpb*. *pentosus* O17 inoculum (0 days for trial B; after 7 days for trial C). **S**, content and timing of salt addition in brine: 7 kg NaCl/100 kg olives (10.5% *w*/*v* NaCl in brine) for trials A (after 35 days) and B (after 25 days); 4 kg NaCl/100 kg olives (6% *w*/*v* NaCl in brine) at time 0 days and subsequent addition of 3 kg NaCl/100 kg olives (4.5% *w*/*v* NaCl in brine) after 25 days for trial C.

Yeast populations in both brines and olives were mainly affected by incubation time rather than the addition of salt and the starter culture. No differences were detected during the overall fermentation period (Figure 6). *Enterobacteriaceae* was affected by fermentation type; the presence of the starter culture and the addition of salt significantly impaired their growth in brine, leading to a complete disappearance after 30 days of fermentation. *Enterobacteriaceae* were not detected on the olives surfaces, probably due to their poor adhesion capability (Figure S2). Overall, a low total bacteria count was found in brines and on the olives surfaces during the early stage of fermentation (0–25 days for trials B and C; 0–35 days for trial A). The addition of salt completely inhibited the growth of mesophilic bacteria, and no growth was detected until the end of fermentation.

Regarding the halophilic microorganisms, a significant increase was observed only for the yeast fraction (Figure S3). In trial C, halophilic yeasts developed already in the first month of fermentation, while in trials A and B, their growth started after salt addition (Figure S3). In contrast, halophilic LAB and other bacteria were not detected on the selective medium used. At the end of fermentation, *Listeria monocytogenes* and *Staphylococcus aureus* were not detected, indicating that fermentation conditions were inhibitory to the development of pathogenic microorganisms.

The LAB population in the spontaneous fermentation was affected by several factors (e.g., salt content, poor energy sources, natural inhibitory compounds), and the trend observed in this study is consistent with previous findings [2,33,34,35].

Several authors [33,36,37,38,39] have reported that spontaneous fermentations were driven by LAB, mainly belonging to the species *Lpb*. *plantarum*, *Lpb*. *paraplantarum*, *Lpb*. *pentosus* and, to a lesser extent, *Lcb*. *casei*. However, these spontaneous processes are not fully predictable due to the possible presence of undesired microorganisms [36,40,41]; therefore, the exploitation of suitable lactobacilli as starter cultures may improve the control and management of the fermentative process [8,14,30,31,32,33,42,43,44,45,46]. Among LAB, *Lpb*. *plantarum* and *Lpb*. *pentosus* are widely recognized as key starters in the fermentation of table olives due to their tolerance to saline and acidic environments, effective acidification kinetics, and positive role on both microbiological quality and sensory attributes [3,6]. In this study, *Lpb*. *pentosus* O17 was selected from a previous screening [15] for its tolerance to phenolic compounds, ability to degrade oleuropein and synthesize hydroxytyrosol, biofilm formation, high radical scavenging activity, and resistance to simulated gastro-intestinal conditions and to different combinations of high NaCl and low pH. Moreover, O17 harbors key genes (i.e., *β*-glucosidase, gallate decarboxylase subunits C, p-coumaric acid decarboxylase, esterase, tannase subunits A and B, transcriptional regulator *PadR*) involved in phenolic compounds metabolism [15].

The survival of *Lpb*. *pentosus* O17 during olive fermentations was monitored by plate counting on the differential medium mMRS, demonstrating a significant drop in cell viability after salt addition. Many authors [23,28,32,41,43,47,48,49] have reported the poor survival of starter cultures in brines with high salt concentrations (especially in natural fermented olives); on the contrary, when lower amounts of salt were used (e.g., 6% *w*/*v* NaCl), the viability of starter cultures is not affected [33]. Moreover, yeasts associated with the olive surface (e.g., *Leccino*, *Cellina di Nardò*, *Kalamàta* and *Conservolea* cultivars [28,47]), are often more tolerant to osmotic stress and may drive fermentative processes. Our results about microbial dynamics (e.g., low survival of LAB, dominance of yeasts after 30 days of fermentation) were also consistent with data obtained by Sacchi et al. [29] for PDO *Gaeta* olives. In agreement with other studies [45,50,51], the use of starter cultures contributed to a reduction in enterobacteria. The final pH values (≤4.5) of *Gaeta*-like olives inhibited the growth of acid-sensitive bacteria, preventing spoilage and ensuring microbial safety during both fermentation and storage [14,28,29,38,47,49].

**Figure 7:**
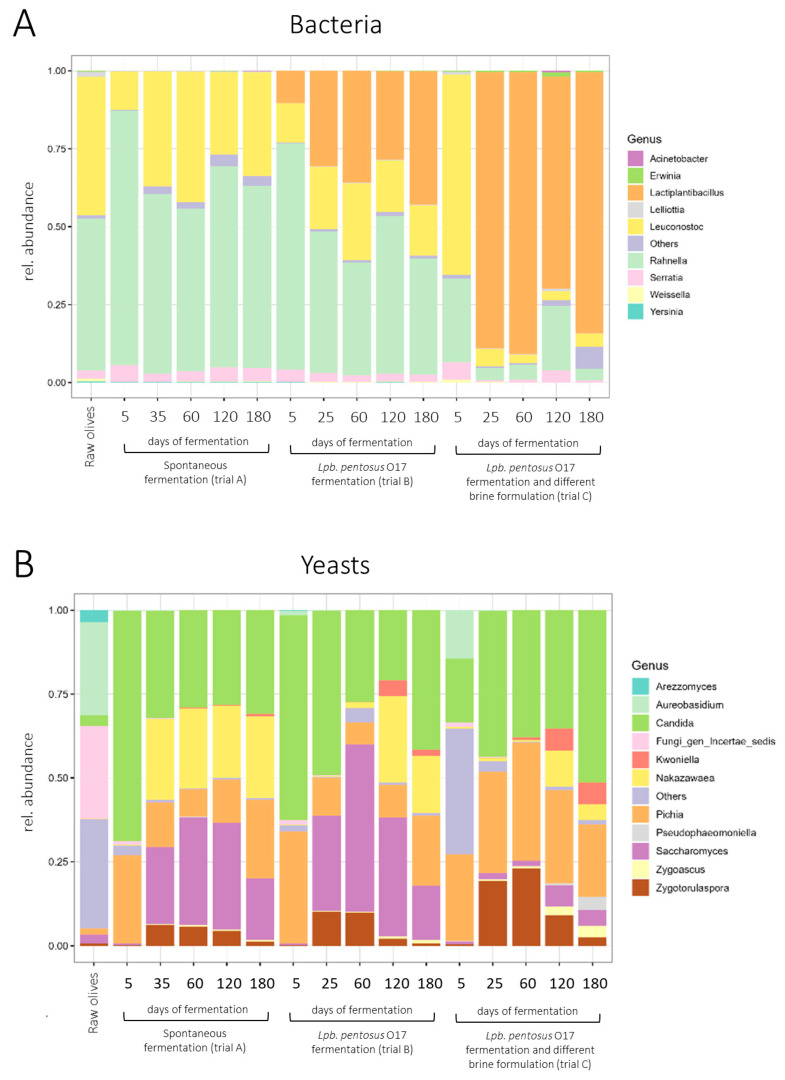
Evolution (expressed as relative abundance) of bacterial (V3–V4 region of *16S rRNA* gene; box (**A**)) and yeast/fungal (*ITS2* region; box (**B**)) microbiota in raw olives (before fermentation process) and brines undergoing spontaneous (trial A) and driven processes (trials B and C), collected at different times of fermentation.

The evolution of bacterial (V3–V4 region of *16S rRNA* gene) and yeasts/fungal (*ITS2* region) microbiota in raw *Itrana* olives (before fermentation process) and brines undergoing spontaneous (trial A) and driven processes (trials B and C), collected at different times of fermentation (i.e., 5, 35, 60, 120, 180 days of fermentation), was, respectively, reported in Figure 7A,B. The relative abundance of the main bacteria and yeast genera was retrieved from the FoodMicrobionet 5.0.1 database (study code ST253 [19]). The bacterial microbiota (Figure 7A) of unprocessed olives (control samples) mainly consisted of the *Rahnella* (49%) and *Leuconostoc* (44%) genera; a very low percentage of *Serratia* (3%), *Lelliottia* (2%) and *Weissella* (1%) were also found (Figure 7A). A similar composition was observed in trial A throughout the fermentation period (Figure 7A). As expected, the genus *Lactiplantibacillus* co-occurred in trial B (unlike the results of plate counting) together with *Rahnella* and *Leuconostoc* (Figure 7A), and completely dominated trial C after 35 days of fermentation (also in this case, unlike what was detected by plate counting), causing a significant decrease in the above-mentioned species. *Lpb. pentosus* O17, being tolerant to osmotic stress, probably survived to a greater extent than the indigenous microbiota of trial C.

Some discrepancies between plate counting and metataxonomic data are probably due to the possible persistence of DNA from dead cells; the inability to distinguish living and dead cells, in fact, is a constraint of DNA-based sequencing [52]. However, the low levels of viability detected by plate counting could also be related to the presence of viable but non-culturable cells (VBNC), not necessarily dead ones. The VBNC state, in fact, is a survival strategy of many microorganisms in response to harsh conditions, including osmotic stress and high salinity [53]. In this study, however, no recovery medium to distinguish VBNC from dead cells was used; therefore, the cause of this discrepancy could not be determined. Similar evidence was also reported by Parente et al. [54].

The mycobiota of unprocessed olives (mainly belonging to the genus *Aureobasidium*, or fungi with *incertae sedis*) were clearly different from those of brines (Figure 7B). A similar composition was observed in the early stage of fermentation (5 days) for trials A and B, with a significant occurrence of *Candida* (68% in trial A, 61% in trial B) and *Pichia* (26% in trial A, 33% in trial B); while a lower abundance of *Candida* (18%) was found in trial C, probably due to the early salt addition (Figure 7B). Mycobiota variability increased during spontaneous fermentation (trial A), with the clear occurrence (beside *Candida* and *Pichia*) of *Nakazawaea* (23% in the middle-stage of fermentation, i.e., 35–60 days; 21–24% in the late-stage of fermentation, i.e., 120–180 days), *Saccharomyces* (23–32% in the middle-stage; 18–31% in the late-stage) and *Zygotorulaspora* (5–6% in the middle-stage; 1–4% in the late-stage) genera, throughout the process (Figure 7B). In trial B, *Nakazawaea* occurred only after 120 days of fermentation. The fungal pattern of trial C was dominated by *Candida* (38%), *Pichia* (35%) and *Zygotorulaspora* (23%) in the middle stage of fermentation (25–60 days), while it became more complex, with the appearance of *Kwoniella* (6%) and *Zygoascus* (3%), in the later stage of the process (120–180 days, Figure 7B). These results demonstrated that both the starter culture (*Lpb. pentosus* O17) and the different brine formulations significantly affected the evolution of indigenous microbiota during *Gaeta*-like olive fermentation.

### 3.3. Mechanical and Sensory Analyses of Gaeta-like Olives

The Texture Profile Analysis (TPA) was carried out to investigate the effect of fermentation on rheological properties of flesh, and differences among olive samples in terms of hardness, cohesiveness, gumminess and chewiness parameters were found (Table 1). Olives from trial C exhibited the lowest flesh hardness, gumminess and chewiness values compared to those obtained through the spontaneous process (trial A) and fermentation driven by *Lpb. pentosus* O17 (trial B). These differences were probably related to the different brine formulation used in trial C (see Figure 1), which affected salt diffusion from the brine to the olives. Furthermore, olives from trial B showed the highest values of the hardness, gumminess and chewiness attributes (Table 1). All *Gaeta*-like olives showed an absence of adhesiveness, and no difference was observed for springiness. Few studies have addressed the rheological properties of table olives [20,55,56], as texture may differ among cultivars and may be strongly affected by fermentation methods. The production process did not affect olive peel break force. Despite the values among samples slightly varying, ranging between 1.7 and 2.1 N, no statistical differences were observed (Figure S4). In this study, *Gaeta*-like olives fermented under early brining conditions (trial C) exhibited lower flesh firmness, probably due to the different salt diffusion from brine to olive flesh. Fadda et al. [55], on the other hand, did not highlight significant differences in the flesh hardness of green olives fermented in brine containing 4% or 7% *w*/*v* NaCl, although a significant decrease in firmness was observed over 180 days of fermentation, probably because of loss of turgor and degradation of pectic polysaccharides.

The sensory profiles of fermented olives, obtained through Quantitative Descriptive Analysis (QDA), are reported in Figure S5. Significant differences (*p* < 0.01) among trials were observed for the attributes “skin hardness”, “ease of stone detachment from flesh” and “bitterness”. *Gaeta*-like olives obtained from trial C were characterized by greater skin hardness and a higher perception of the bitterness attribute (Figure S5) compared to those obtained in fermentative processes A and B. These differences were probably related to the different brine formulation used in trial C (see Figure 1). Some authors [57,58], in fact, have demonstrated that high salt concentrations may inhibit β-glucosidase activity in both enzymatic model systems and olive fermentation conditions, probably due to ionic-strength-induced perturbations of catalytic residues and enzyme conformation. Since β-glucosidases are involved in oleuropein hydrolysis during olive fermentation, early salt addition may have reduced the enzymatic activity and consequently the oleuropein degradation, resulting in a higher perception of bitterness. Olives from trial A showed a higher ease of stone detachment from flesh (Figure S5).

In contrast with these results (lower intensity of the “bitter” attribute for trial-A-PDO *Gaeta* olives and trial-B-*Gaeta*-like olives), Sacchi et al. [29] reported a higher perception of both “bitter” and “sour” attributes in *Gaeta* table olives fermented for 30 days in water followed by the addition of salt (6% and 8% *w*/*v*). This difference was likely due to the impact of salt supplementation on microbiological acidification and debittering dynamics.

The composition of microbial communities may also affect the metabolite production and sensory properties of fermented table olives [3,59]. In this study, direct correlation of sensory attributes and bacterial/yeast/fungal microbiota was rather complex, since we did not perform detailed metabolomic analyses (e.g., CG-MS, UPLC-MS), which could have provided in-depth information about the relevant metabolites produced during fermentation. Additionally, as in other fermented foods, changes in organoleptic properties are the result of complex metabolic interactions that occur among the members of a microbial community; therefore, more detailed metabolic studies are needed.

## 4. Conclusions

The performance and efficiency of starter cultures are strain-specific; therefore, the selection of suitable strains is crucial for driving high-quality fermentative processes. In this study, we used *Lpb*. *pentosus* O17 as a starter culture for the production of *Gaeta*-like olives, as it possessed several features (such as osmotic and acidic tolerance, phenolic compound resistance, and high radical scavenging activity) useful for driving olive fermentation and obtaining a high-quality product.

*Lpb. pentosus* O17 led to faster brine acidification, improving the microbiological quality of the final product, compared to the PDO-based process. As indicated by metataxonomic results, *Lpb. pentosus* O17 may promote the dominance of beneficial microorganisms while reducing the occurrence of spoilage-associated taxa. Moreover, the early addition of salt (as in trial C), which was not even foreseen by the PDO regulation, could modulate the composition of indigenous microbiota and, therefore, could be a further strategy to reduce the development of undesired microorganisms right from the early stages of fermentation. To date, few studies have investigated the use of starter cultures for the production of *Gaeta*-like olives. Our study provides further insights into both starter culture performance and its potential application to improve the quality and reproducibility of this process. Moreover, we suggested that the use of starter cultures and a different brining method could improve the microbiological quality of *Gaeta*-like olives without altering the organoleptic properties related to the PDO product.

## Figures and Tables

**Table 1:** Texture Profile Analysis of *Gaeta*-like olives at the end of 180-day-long fermentation.

Sample ^1^	Hardness ^2^	Springiness ^2^	Cohesiveness ^2^	Gumminess ^2^	Chewiness ^2^
**Trial A**	12.68 ± 3.92 ^a^	0.78 ± 0.08 ^a^	0.36 ± 0.07 ^a^	4.46 ± 1.30 ^a^	3.49 ± 1.12 ^a^
**Trial B**	15.49 ± 3.98 ^b^	0.75 ± 0.09 ^a^	0.34 ± 0.08 ^a^	5.10 ± 1.26 ^a^	3.85 ± 1.21 ^a^
**Trial C**	10.36 ± 3.03 ^c,^*	0.80 ± 0.07 ^a,^*	0.39 ± 0.07 ^a,^*	3.97 ± 0.99 ^a,^*	3.16 ± 0.80 ^a,^*

Mean values ± standard deviations are reported. ^1^ Trial A: spontaneous fermentation; Trial B: driven fermentation using *Lpb. pentosus* O17 as a starter culture; Trial C: driven fermentation using *Lpb. pentosus* O17 as a starter culture and different brine formulation. ^2^ Hardness was expressed as Newton; Springiness, mm; Cohesiveness, mm^2^; Gumminess, hardness × cohesiveness; Chewiness, gumminess × springiness. For each parameter, letters (a, b, c) indicate significant difference (Tukey’s HSD, *p* ≤ 0.01) compared to trials A (spontaneous fermentation; reference condition); * indicates significant difference (Tukey’s HSD, *p* ≤ 0.01) between driven fermentations (trial B and trial C).

## Data Availability

The original contributions presented in this study are included in the article/Supplementary Material. Further inquiries can be directed to the corresponding author.

## Supplementary Materials

The following supporting information can be downloaded at https://www.mdpi.com/article/10.3390/foods15071257/s1: Table S1: Physicochemical parameters measured on olives and brines at the end of fermentation (180 days); Figure S1: Changes in titratable acidity measured in olives (continuous lines) and brines (dotted lines) during spontaneous (trial A, red line) and driven fermentations (trial B, green line, *Lpb. pentosus* O17 inoculated in water at the beginning of the process; trial C, blue line, *Lpb. pentosus* O17 inoculated after 7 days of fermentation in 6% *w*/*v* brine solution). **I**, timing of *Lpb. pentosus* O17 inoculum (0 days for trial B; after 7 days for trial C). **S**, content and timing of salt addition in brine: 7 kg NaCl/100 kg olives (10.5% *w*/*v* NaCl in brine) for trials A (after 35 days) and B (after 25 days); 4 kg NaCl/100 kg olives (6% *w*/*v* NaCl in brine) at time 0 days and subsequent addition of 3 kg NaCl/100 kg olives (4.5% *w*/*v* NaCl in brine) after 25 days for trial C; Figure S2: Enterobacteria population measured in olives (continuous lines) and brines (dotted lines) during spontaneous (trial A, red line) and driven fermentations (trial B, green line, *Lpb. pentosus* O17 inoculated in water at the beginning of the process; trial C, blue line, *Lpb. pentosus* O17 inoculated after 7 days of fermentation in 6% *w*/*v* brine solution). **I**, timing of *Lpb. pentosus* O17 inoculum (0 days for trial B; after 7 days for trial C). **S**, content and timing of salt addition in brine: 7 kg NaCl/100 kg olives (10.5% *w*/*v* NaCl in brine) for trials A (after 35 days) and B (after 25 days); 4 kg NaCl/100 kg olives (6% *w*/*v* NaCl in brine) at time 0 days and subsequent addition of 3 kg NaCl/100 kg olives (4.5% *w*/*v* NaCl in brine) after 25 days for trial C; Figure S3: Halophilic yeasts population measured in olives (continuous lines) and brines (dotted lines) during spontaneous (trial A, red line) and driven fermentations (trial B, green line, *Lpb. pentosus* O17 inoculated in water at the beginning of the process; trial C, blue line, *Lpb. pentosus* O17 inoculated after 7 days of fermentation in 6% *w*/*v* brine solution). **I**, timing of *Lpb. pentosus* O17 inoculum (0 days for trial B; after 7 days for trial C). **S**, content and timing of salt addition in brine: 7 kg NaCl/100 kg olives (10.5% *w*/*v* NaCl in brine) for trials A (after 35 days) and B (after 25 days); 4 kg NaCl/100 kg olives (6% *w*/*v* NaCl in brine) at time 0 days and subsequent addition of 3 kg NaCl/100 kg olives (4.5% *w*/*v* NaCl in brine) after 25 days for trial C; Figure S4: Boxplot showing the olive peel break force (Newton, N) in trial A (spontaneous fermentation), trial B (driven fermentation using *Lpb. pentosus* O17 as starter culture), trial C (driven fermentation using *Lpb. pentosus* O17 as starter culture and different brine formulation); Figure S5: Sensory profile of *Gaeta*-like olives at the end of 180-day-long fermentation. Letters indicate significant difference (LSD test, *p* ≤ 0.01) among different trials (trial A vs. B vs. C); attributes without letters are not significant.

## Abbreviations

The following abbreviations are used in this manuscript:

LAB	Lactic Acid Bacteria
PDO	Protected Designation of Origin
