# Supplementary material for: Use of Lactiplantibacillus pentosus O17 as a Starter Culture for the Production of Gaeta-like Table Olives
Source: Foods. 2026 Apr 7;15(7):1257. doi: 10.3390/foods15071257 (PMC13073220; doi:10.3390/foods15071257)
Supplement: Supplementary file 1 [file foods-15-01257-s001.zip › Supplementary Materials_paper_O17_rev_ok.pdf]

Title

Use of *Lactiplantibacillus pentosus* O17 as a starter culture for the production of *Gaeta*-like table olives

Authors

Marilisa Giavalisco *, Annamaria Ricciardi, Emanuela Lavanga, Attilio Matera, Nicola Condelli and Teresa Zotta

Affiliations

Department of Agricultural, Forestry, Food and Environmental Sciences, University of Basilicata, 85100 Po-tenza, Italy; marilisa.giavalisco@unibas.it (M.G.); annamaria.ricciardi@unibas.it (A.R.); emanuela.lavanga@unibas.it (E.L); attilio.matera@unibas.it (A.M); nicola.condelli@unibas.it (N.C.); teresa.zotta@unibas.it (T.Z.).

* Correspondence: marilisa.giavalisco@unibas.it; Tel +39-0971-205576

Table S1. Physicochemical parameters measured on olives and brines at the end of fermentation (180 days).

Sample	pH		Titratable acidity (% of lactic acid)		Salt content (% of NaCl)		Total Phenolic Content (g GAE/kg)		Moisture content	Water activity
	Olives	Brine	Olives	Brine	Olives	Brine	Olives	Brine		
**Fresh olives**	5.38±0.00*	-	0.05±0.00*	0	0	0	44.85±0.26*	0	59.43±0.89[a]	0.97±0.00*
**Trial A**	5.03±0.04[a]	4.50±0.01[a]	0.03±0.00[a]	0.28±0.01[a]	2.05±0.45[a]	8.37±0.12[a]	16.64±0.83[a]	21.00±0.88[a]	58.08±1.6[a]	0.97±0.00*,[a]
**Trial B**	5.02±0.01[a]	4.50±0.01[a]	0.03±0.00[a]	0.25±0.01[b]	2.17±0.44[a]	8.36±0.12[a]	15.02±1.24[a]	17.18±0.50[b]	61.55±1.8[a]	0.98±0.00[b]
**Trial C**	5.12±0.02[b]	4.50±0.00[a]	0.03±0.00[a]	0.23±0.01[b]	1.78±0.85[b]	8.67±0.04[b]	16.54±1.12[a]	18.90±0.75[b]	58.94±2.6[a]	0.98±0.00[b]

Mean values ± standard deviations are reported. Fresh olives: unprocessed olives of *Itrana* cultivar used for the fermentation; Trial A: spontaneous fermentation; Trial B: driven fermentation using *Lpb. pentosus* O17 as starter culture; Trial C: driven fermentation using *Lpb. pentosus* O17 as starter culture and different brine formulation. Symbol (*) indicate significant difference (Tukey's HSD, $p \leq 0.001$) among time (t0 *vs* t180); letters indicate significant difference (Tukey's HSD, $p \leq 0.001$) among different 180-days-brines (Trial A *vs* B *vs* C).

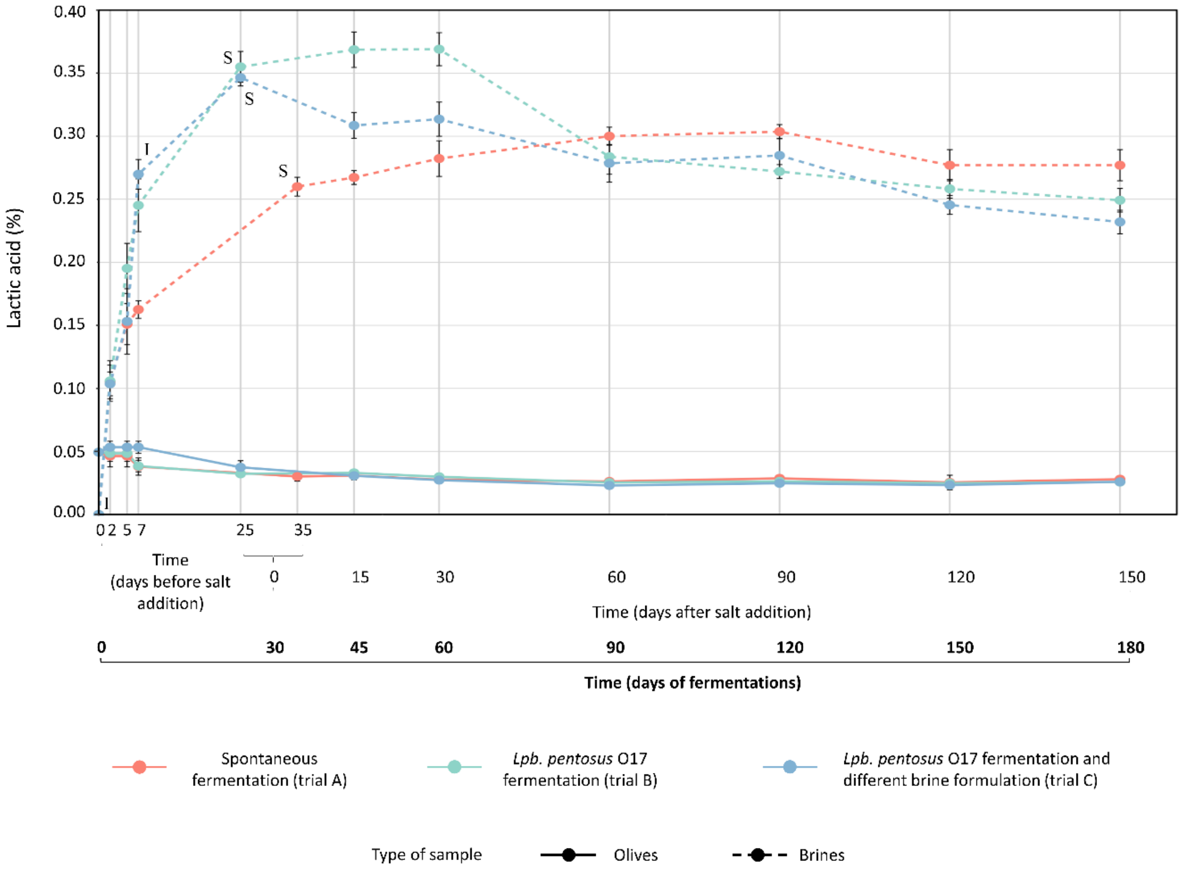

Supplementary Figure S1. Changes in titratable acidity measured in olives (continuous lines) and brines (dotted lines) during spontaneous (trial A, red line) and driven fermentations (trial B, green line, *Lpb. pentosus* O17 inoculated in water at the beginning of the process; trial C, blue line, *Lpb. pentosus* O17 inoculated after 7 days of fermentation in 6% *w/v* brine solution). **I**, timing of *Lpb. pentosus* O17 inoculum (0 days for trial B; after 7 days for trial C). **S**, content and timing of salt addition in brine: 7 kg NaCl/100 kg olives (10.5% *w/v* NaCl in brine) for trials A (after 35 days) and B (after 25 days); 4 kg NaCl/100 kg olives (6% *w/v* NaCl in brine) at time 0 days and subsequent addition of 3 kg NaCl/100 kg olives (4.5% *w/v* NaCl in brine) after 25 days for trial C.

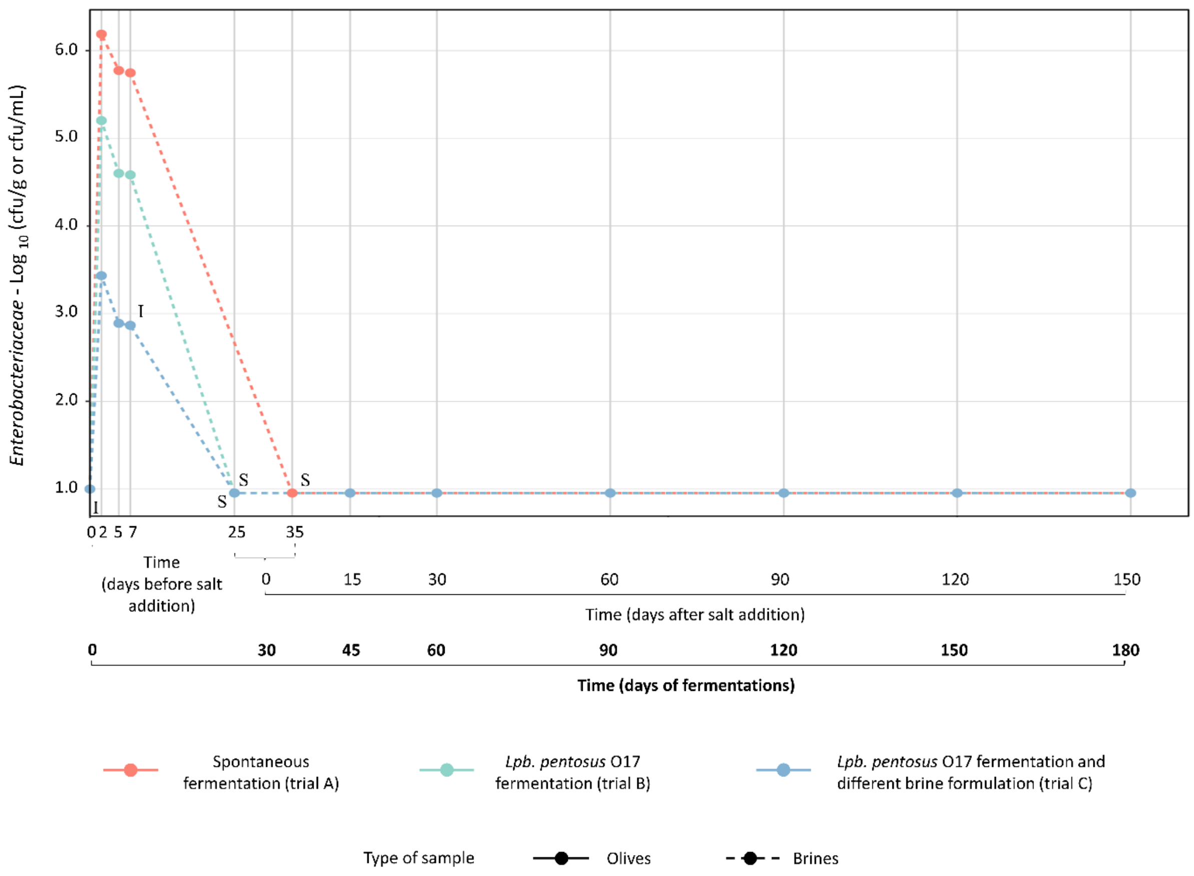

Supplementary Figure S2. Enterobacteria population measured in olives (continuous lines) and brines (dotted lines) during spontaneous (trial A, red line) and driven fermentations (trial B, green line, *Lpb. pentosus* O17 inoculated in water at the beginning of the process; trial C, blue line, *Lpb. pentosus* O17 inoculated after 7 days of fermentation in 6% *w/v* brine solution). **I**, timing of *Lpb. pentosus* O17 inoculum (0 days for trial B; after 7 days for trial C). **S**, content and timing of salt addition in brine: 7 kg NaCl/100 kg olives (10.5% *w/v* NaCl in brine) for trials A (after 35 days) and B (after 25 days); 4 kg NaCl/100 kg olives (6% *w/v* NaCl in brine) at time 0 days and subsequent addition of 3 kg NaCl/100 kg olives (4.5% *w/v* NaCl in brine) after 25 days for trial C.

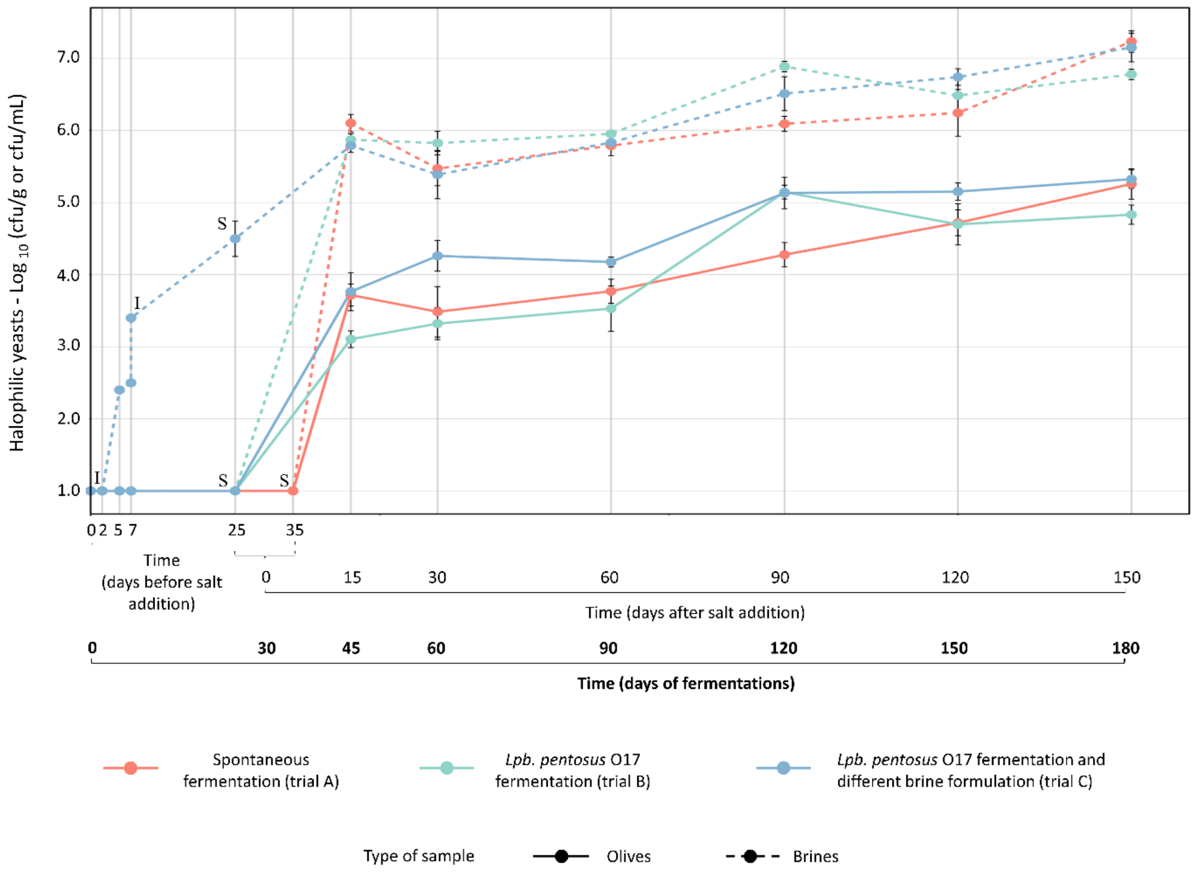

Supplementary Figure S3. Halophilic yeasts population measured in olives (continuous lines) and brines (dotted lines) during spontaneous (trial A, red line) and driven fermentations (trial B, green line, *Lpb. pentosus* O17 inoculated in water at the beginning of the process; trial C, blue line, *Lpb. pentosus* O17 inoculated after 7 days of fermentation in 6% *w/v* brine solution). **I**, timing of *Lpb. pentosus* O17 inoculum (0 days for trial B; after 7 days for trial C). **S**, content and timing of salt addition in brine: 7 kg NaCl/100 kg olives (10.5% *w/v* NaCl in brine) for trials A (after 35 days) and B (after 25 days); 4 kg NaCl/100 kg olives (6% *w/v* NaCl in brine) at time 0 days and subsequent addition of 3 kg NaCl/100 kg olives (4.5% *w/v* NaCl in brine) after 25 days for trial C.

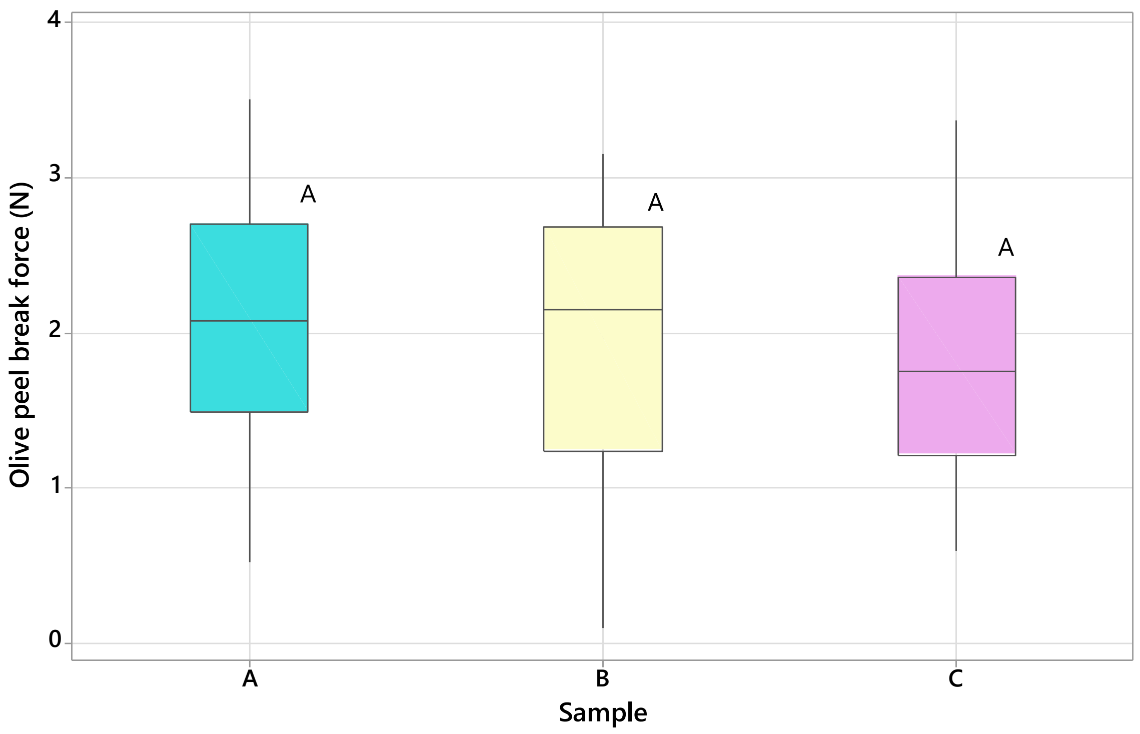

Supplementary Figure S4. Boxplot showing the olive peel break force (Newton, N) in trial A (spontaneous fermentation), trial B (driven fermentation using *Lpb. pentosus* O17 as starter culture), trial C (driven fermentation using *Lpb. pentosus* O17 as starter culture and different brine formulation).

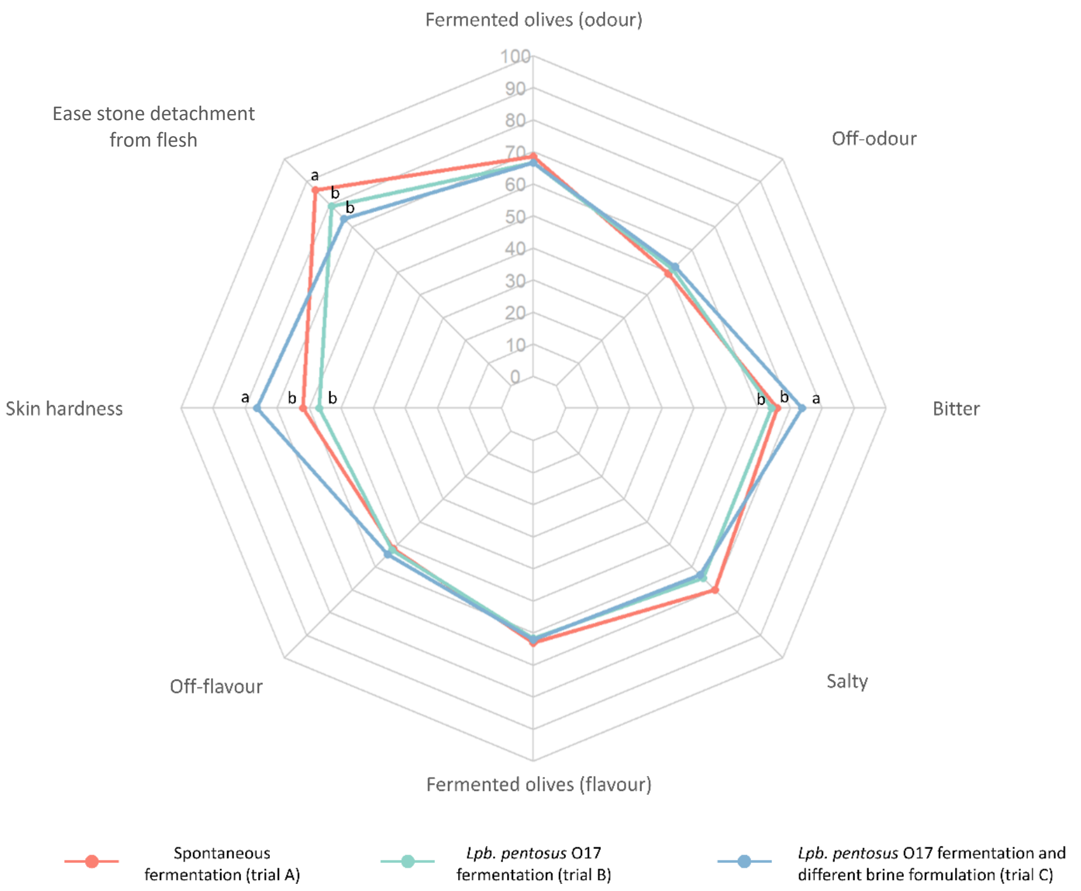

Supplementary Figure S5. Sensory profile of *Gaeta*-like olives at the end of 180-day-long fermentation. Letters indicate significant difference (LSD test, $p \leq 0.01$) among different trials (trial A *vs* B *vs* C); attributes without letters are not significant.
